# A new species of *Tanna* Distant, 1905 from Yunnan, China (Hemiptera, Cicadidae, Cicadinae)

**DOI:** 10.3897/BDJ.12.e115715

**Published:** 2024-01-25

**Authors:** Cheng-Bin Wang

**Affiliations:** 1 Engineering Research Center for Forest and Grassland Disaster Prevention and Reduction, Mianyang Normal University, 166 Mianxing West Road, Mianyang, China Engineering Research Center for Forest and Grassland Disaster Prevention and Reduction, Mianyang Normal University, 166 Mianxing West Road Mianyang China

**Keywords:** Cicada, Leptopsaltriini, taxonomy, morphology, Oriental Region

## Abstract

**Background:**

The genus *Tanna* Distant, 1905 (Hemiptera, Cicadidae, Cicadinae, Leptopsaltriini, Leptopsaltriina) currently includes 23 species (three tentatively placed), with its actual geographical distribution in China, Japan, Nepal, Bhutan, Cambodia, Thailand and Vietnam. Most of them, 16 species, are known from China, including one new species here described.

**New information:**

A new species of cicada, *Tannafengi* Wang **sp. nov.**, is described from Yunnan, southwest China. Colour plates are presented to illustrate all diagnostic characters. An updated list of *Tanna* species occurring in China is provided.

## Introduction

Since the publication of the milestone book on Chinese cicadas, "The Cicadidae of China (Homoptera: Cicadoidea)" (Chou et al. 1997), it has become an essential source for Chinese cicada researchers and amateurs. However, content of the genus *Tanna* Distant, 1905 and other relative genera, is disarrayed and rough to interpret. Seven species were included of *Tanna*: *T.apicalis* Chen, 1940, *T.herzbergi* Schmidt, 1932, *T.japonensis* (Distant, 1892), *T.ornata* Kato, 1940, *T.pseudocalis* Lei & Chou, 1997, *T.taikosana* Kato, 1925 and *T.taipinensis* Mstsumura, 1907. Amongst them, yet only *T.ornata* Kato, 1940 and *T.taipinensis* Mstsumura, 1907 are valid or still retained within *Tanna*. For *T.japonensis* (Distant, 1892) which is restricted to Japan and Amami Ôshima, it was misidentified as some Chinese species. Moreover, seven species were missing in Chou et al. (1997): *T.auripennis* Kato, 1930, *T.chekiangensis* Ôuchi, 1938, *T.karenkonis* Kato, 1939, *T.ornatipennis* Esaki, 1933, *T.sayurie* Kato, 1926, *T.sozanensis* Kato, 1926 and *T.tairikuana* Kato, 1940.

Later, *T.taikosana* Kato, 1925 and *T.horiensis* Kato, 1926 were synonymised with *T.taipinensis* Mstsumura, 1907 and *T.infuscata* Lee & Hayashi, 2004 was described from Taiwan (Lee and Hayashi 2004). *Tannaaquilonia* Lee & Lei, 2014 was described from Xinjiang and *Neotanna* Kato, 1927 was treated as a junior synonym of *Tanna*; correspondingly, *N.abdominalis* Kato, 1938 and *N.viridis* Kato, 1925 were transferred to *Tanna* (Lee and Lei 2014). Lee (2016) proposed the monotypic genus *Galgoria* Lee, 2016 to accommodate *T.herzbergi* Schmidt, 1932; meanwhile, he treated *T.apicalis* Chen, 1940 and *T.pseudocalis* Lei & Chou, 1997 as synonyms of *G.herzbergi* (Schmidt, 1932). Recently, *T.bhutanensis* Distant, 1912 was recorded from south China and *T.obliqua* Liu, 1940 was resurrected from synonymy of *T.auripennis* Kato, 1930 (synonymised by Kato (1956)) (Lee and Emery (2020)).

The present study on *Tanna* material from Yunnan Province of China (representing a new provincial record for the genus) yielded one new species to science, where all diagnostic characters are broadly illustrated. Nowadays, 16 species of the genus *Tanna* are known from China, including *T.fengi* Wang **sp. nov.** here described below.

### Species list of Tanna Distant, 1905 from China

(Alphabetically listed and modified from Sanborn (2013) and Lee and Emery (2020)).

1. *Tannaabdominalis* (Kato, 1938) (= *Neotannaabdominalis* = *Neotannasinensis* Ôuchi, 1938 = *Neotannasimultaneous* Chen, 1940) (Anhui, Fujian, Guangxi, Hunan, Zhejiang);
2. *Tannaaquilonia* Lee & Lei, 2014 (Xinjiang);
3. *Tannaauripennis* Kato, 1930 (Taiwan);
4. *Tannabhutanensis* Distant, 1912 (= *Neotannabhutanensis*) (S. China; Bhutan, Nepal);
5. *Tannachekiangensis* Ôuchi, 1938 (= *Tannasinensis* Kato, 1938 = *Tannajaponensis* (nec Distant)) (Anhui, Fujian, Zhejiang);
6. *Tannafengi* Wang **sp. nov.** (Yunnan);
7. *Tannainfuscata* Lee & Hayashi, 2004 (= *Tannainfuscate* (sic)) (Taiwan);
8. *Tannakarenkonis* Kato, 1939 (Taiwan);
9. *Tannaobliqua* Liu, 1940 (Fujian, Hunan, Jiangxi, Sichuan, Zhejiang);
10. *Tannaornata* Kato, 1940 (Zhejiang);
11. *Tannaornatipennis* Esaki, 1933 (= *Tanna (Neotanna) ornatipennis = Neotanna ornatipennis*) (Taiwan);
12. *Tannasayurie* Kato, 1926 (Taiwan);
13. *Tannasozanensis* Kato, 1926 (Taiwan);
14. *Tannataipinensis* (Matsumura, 1907) (= *Leptopsaltriataipinensis* = *Tannatapinensis* (sic) = *Tannataikosana* Kato, 1925 = *Tannahoriensis* Kato, 1926) (Taiwan);
15. *Tannatairikuana* Kato, 1940 (Guangxi, Sichuan);
16. *Tannaviridis* Kato, 1925 (= *Neotannaviridis* = *Neottanna* (sic) *viridis* = *Tannahorishana* Kato, 1925 = *Neotannahorishana* = *Neotannahorishaua* (sic) = *Euterpnosiahorishana* = *Tannaviridisniitakaensis* Kato, 1926 = *Neotannaviridisniitakaensis* = *Neotannatarowanensis* Matsumura, 1927 = *Neotannarantaizana* Matsumura, 1927 = *Neotannaviridisinmaculata* Kato, 1932 = *Tannaviridisimmaculata* (sic)) (Taiwan).

## Materials and methods

Fieldwork recovered six males and eight females of the new species from Yunnan of China in July 2022 and were kept in cold fluid (-20℃). Specimens were relaxed and softened in water at room temperature for 24 hours and then placed in distilled water for cleaning and dissection. To examine the male genitalia, the pygofer together with sternite VIII were detached and treated with a 10% potassium hydroxide (KOH) solution at room temperature for 12 hours. They were then placed in distilled water to remove any remaining KOH and prevent further bleaching. After examination, the body parts were mounted on a slide using Euparal Mounting Medium for future studies. Images were taken with a Canon macro photo lens MP-E 65 mm on a Canon 5DsR. Images of the same object at different focal planes were combined using Zerene Stacker 1.04 stacking software. Adobe Photoshop CS6 was used for image post-processing. Description was based on dry specimens. Morphological terminology follows Moulds (2005) and Moulds (2012) and higher taxonomy follows Marshall et al. (2018) and Simon et al. (2019). Measurement criteria in millimetres (mm) follow Wang and Liu (2022).

Type material is deposited in the following institutional and private collections: **MYNU** Invertebrate collection of Mianyang Normal University, Mianyang, China; **cLFW** private collection of Lei Feng, Weifang, China.

## Taxon treatments

### Tanna fengi

Wang sp. nov.

537F7ADC-ACEF-5C40-9B81-B53007389FF6

811D6E10-BC0C-496C-BF19-9A94370A4C8C

#### Materials

- **Type status:** Holotype. **Occurrence:** recordedBy: Xue-Zhou Li; sex: male; occurrenceID: 569FDAB6-84AD-5D0E-96EE-8E048C5B0CB9; **Taxon:** scientificName: *Tannafengi* Wang; **Location:** country: CHINA; stateProvince: Yunnan; verbatimLocality: Diqing Prefecture, Weixi County, Weideng Township, Xinhua Village [新化村]; **Event:** verbatimEventDate: 28.VII.2022; **Record Level:** institutionCode: MYNU
- **Type status:** Paratype. **Occurrence:** recordedBy: Xue-Zhou Li; sex: 3 females; occurrenceID: ADCCBA98-CDD4-5715-A81B-0747F90C4576; **Taxon:** scientificName: *Tannafengi* Wang; **Location:** country: CHINA; stateProvince: Yunnan; verbatimLocality: Diqing Prefecture, Weixi County, Weideng Township, Xinhua Village [新化村]; **Event:** verbatimEventDate: 28.VII.2022; **Record Level:** institutionCode: MYNU
- **Type status:** Paratype. **Occurrence:** recordedBy: Xue-Zhou Li; sex: 5 males, 5 females; occurrenceID: F4AA317F-48D1-5E46-B7EC-054A1470276C; **Taxon:** scientificName: *Tannafengi* Wang; **Location:** country: CHINA; stateProvince: Yunnan; verbatimLocality: Diqing Prefecture, Weixi County, Weideng Township, Xinhua Village [新化村]; **Event:** verbatimEventDate: 28.VII.2022; **Record Level:** collectionCode: cLFW

#### Description

**Figure 1.**
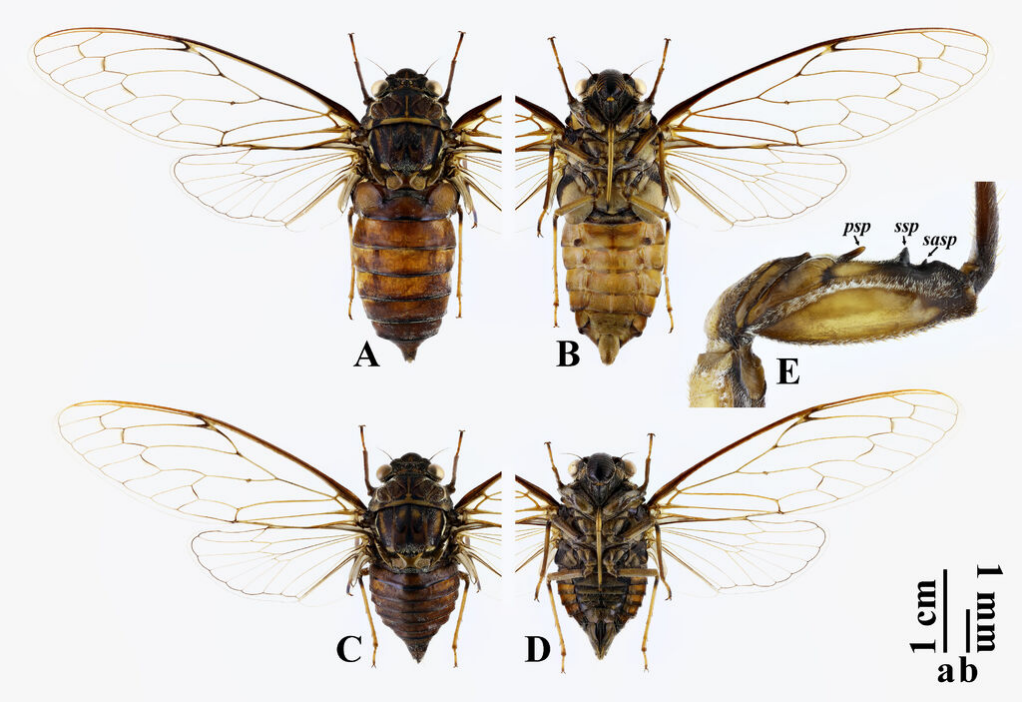
*Tannafengi* Wang **sp. nov.**: **A, B** habitus, holotype, ♂; **C, D** habitus, paratype, ♀; **E** fore femur, holotype, ♂. **A, C** dorsal views; **B, D** ventral views; **E** lateral view. Abbreviations: psp: primary spine; sasp: subapical spine; ssp: secondary spine. Scale bar: a for A–D; b for E.

**Male** (Fig. 1A, B). Measurements (mm, n = 6). Body 33.8–36.1 long. Lengths of different body parts: head (2.3–2.7), pronotum (4.0–4.6), mesonotum (7.5–8.0), forewing (34.6–35.9), abdomen (20.0–20.8); width: head (8.5–8.8), pronotum (10.8–11.2), mesonotum (7.7–8.2), forewing (12.9–13.5). Ratios of different body parts: (pronotal length)/(head length) = 1.8; (mesonotal length excluding cruciform elevation)/(pronotal length) = 1.5; (abdominal length)/(head + pronotal + mesonotal length) = 1.5; (head width)/(pronotal width) = 0.8; (head width)/(mesonotal width) = 1.1; (abdominal tergite III width)/(mesonotal width) = 1.6; (forewing length)/(forewing width) = 2.7.

Head with bottom colour fuscous, with following blackish markings occupying most of head: median fascia enclosing three ocelli, not reaching frontoclypeal suture anteriorly and confluent with posterior fascia posteriorly, with anterior part inverted subtriangular, diverging into two slender stripes at each lateral angle, of which anterior one extending on to and occupying most of supra-antennal plate and posterior one extending longitudinally to lateral ocellus; lateral fasciae rather large, between median fascia and eyes; posterior fascia narrow, along entire posterior margin of head. Compound eyes greyish, protruding. Ocelli brownish. Distance between lateral ocellus and corresponding eye about 2.2 times as wide as distance between lateral ocelli. Antennae fuscous to blackish. Postclypeus distinctly swollen; mostly blackish, with a brownish longitudinal fascia at middle of basal part and brownish between transverse grooves. Anteclypeus mostly blackish, vaguely tinged fuscous near centre and with a brownish spot at base. Genae brownish. Lorum almost entirely blackish. Rostrum brownish, except blackish at apex, reaching posterior margin of abdominal sternite I.

Thorax. Pronotum fuscous at pronotal disc while brownish at pronotal collar, without markings along paramedian and lateral fissures, but with following blackish markings (except brownish median fascia): median fascia slender, roundly dilated at posterior end, far away from anterior margin of pronotum and posteriorly approaching ambient fissure; submedian fasciae long, enclosing median fascia, strongly broadened at both anterior and posterior ends; marginal fasciae relatively wide, along lateral margins of pronotal collar and confluent with front corner spots; front and back corner spots situated before and after hind corners of pronotal collar, respectively; posterior fascia very narrow, along middle part of posterior margin of pronotal collar. Pronotal collar short, ampliate posterolaterally, with lateral margins sinuate, but not dentate; hind corners widely rounded; surface transversely grooved. Mesonotum fuscous, with following blackish markings: median fascia long, relatively slender, somewhat widened posteriorly, reaching anterior margin of cruciform elevation, with posterior part extending laterally to enclose scutal depressions and then curved anteriorly with triangular apices; paramedian fasciae rather wide, occupying most of submedian sigillae, medially curved to join median fascia; accessory fasciae short, slender, between paramedian and lateral fasciae, confluent with lateral fasciae posteriorly; lateral fasciae wide, along medial margins of lateral sigillae, with posterior ends bent laterally and confluent with marginal fasciae; marginal fasciae wide, along lateral margins of mesonotum. Cruciform elevation brown, fuscous to blackish in apical parts of anterior arms. Wing grooves brown. Thoracic sternites brownish, except blackish at katepisterna II.

Legs mostly brownish to brown; femora fuscous to blackish at base; tibiae blackish at base; tarsi fuscous distally. Profemur (Fig. 1E) with three spines: primary spine long, slender and digitiform, obliquely inserted; secondary spine stout, subtrianglular; subapical spine rather small, subtrianglular. Meracanthi mostly blackish.

Wings hyaline. Venation generally fuscous to brownish, more or less lighter apically; R+Sc veins blackish-brown basally. Forewing with 8 apical cells; ulnar cell 3 about 3.0 times as long as apical cell 5; RA_2_ vein with longitudinal portion about 1.7 times as long as basal portion; infuscations distinctly present on r, r-m and m crossveins, slightly on m-cu crossvein and rather inconspicuously on apices of longitudinal veins of apical cells (cannot be seen in photos); nodal line absent; basal cell slightly infuscated; basal membrane brownish. Hindwing with 6 apical cells; jugum and longitudinal margins of vannus brownish.

**Figure 2.**
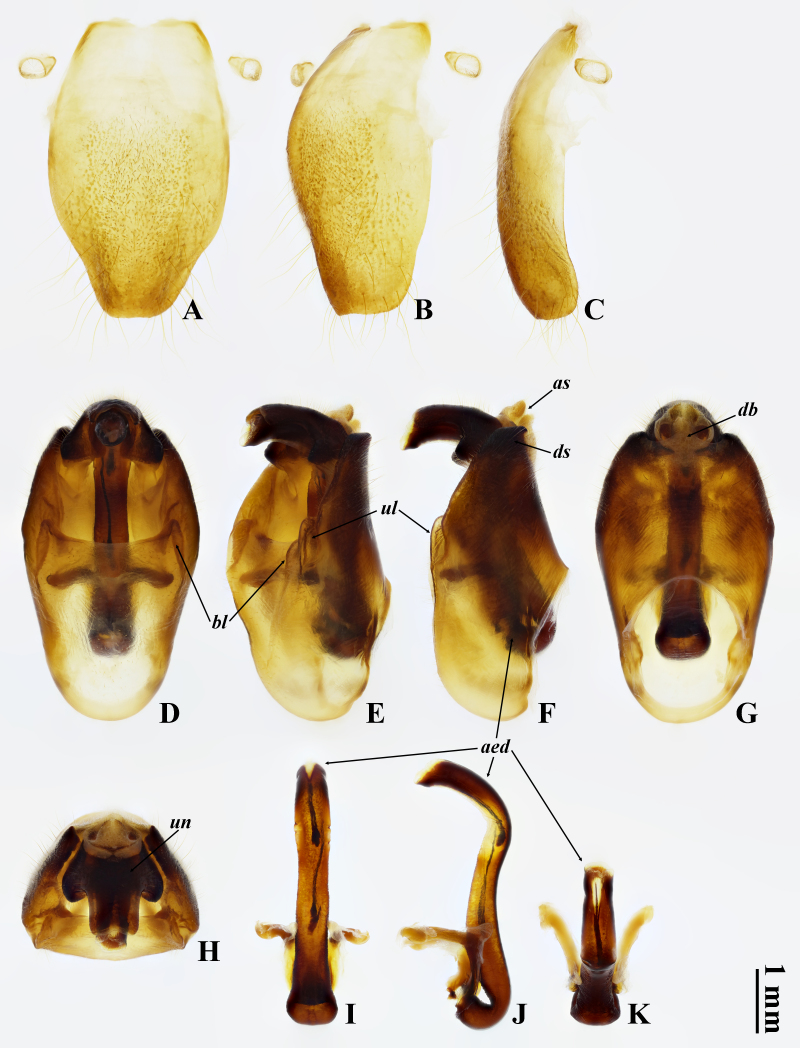
*Tannafengi* Wang **sp. nov.**, holotype, ♂: **A–C** abdominal sternite VIII; **D–H** pygofer; **I–K** aedeagus. **A, D** ventral views; **B, E** ventrolateral views; **C, F, J** lateral views; **G, I** dorsal views; **H, K** apical views. Abbreviations: aed: aedeagus; as: anal styles; bl: basal lobe; db: dorsal beak; ds: distal shoulder; ul: upper lobe; un: uncus.

Abdomen cylindrical, fuscous on dorsum and brownish on venter, with posterior margin of each tergite narrowly blackish, without distinct white pollinosity. Timbal cover scale-like, mostly brown and tinged blackish at medial base, concealing timbal in dorsal view. Operculum mostly brownish, widely blackish along outer margin; scale-like, longer than wide, with medial margin barely emarginate in basal half; apex rounded, extending beyond posterior margin of abdominal sternite II; widely separated from each other with gap as wide as one of them. Abdominal tergites I–III much wider than mesonotum, tergite III 1.6 times as wide as mesonotum. Abdominal sternite III with paired fuscous tubercles near posterolateral corners, protruding posterolaterally; sternite IV with smaller paired brown tubercles around middle of lateral margins, protruding posterolaterally; sternite VII wider than long, gently emarginate in middle of posterior margin; sternite VIII (Fig. 2A–C) oblong, constricting in posterior part, with posterior margin nearly truncate; surface gently convex ventrally; anterolateral apodemes almost absent.

Male genitalia. Pygofer 5.0 mm long and 2.8 mm wide, elliptical in ventral and dorsal views (Fig. 2D, G); anal styles relatively small, less sclerotised, densely covered with short setae apically (Fig. 2F); dorsal beak lightly sclerotised, widely subtriangular in dorsal view (Fig. 2G); basal lobes short, dentiform in ventral view (Fig. 2D) and tuberculate in ventrolateral view (Fig. 2E), abutting to upper lobes; upper lobes longer than basal lobes, digitiform, abutting to side of pygofer (Fig. 2D–F); distal shoulders round in ventral view (Fig. 2D) and obliquely truncate in lateral view (Fig. 2F). Uncus simple, not bifurcate, slender in apical part and gently bent inwards in lateral view (Fig. 2F); apex widely truncate, but emarginate medially (Fig. 2H). Aedeagus thick, slightly narrower than apical part of uncus (Fig. 2I), with apical third strongly curved ventrally in lateral view (Fig. 2J); dorsal surface of apex with an inverted long triangular membranous area (Fig. 2K).

**Female** (Fig. 1C, D). Measurements (mm, n = 8). Body 24.3–26.1 long. Length of different body parts: head (1.8–2.2), pronotum (3.8–4.1), mesonotum (7.6–8.0), forewing (39.4–40.2), abdomen (11.1–11.8); width: head (8.2–8.3), pronotum (10.0–10.3), mesonotum (7.6–7.9), forewing (12.4–12.8). Ratios of different body parts: (pronotal length)/(head length) = 1.7; (mesonotal length excluding cruciform elevation)/(pronotal length) = 1.5; (abdominal length)/(head + pronotal + mesonotal length) = 0.8; (head width)/(pronotal width) = 0.8; (head width)/(mesonotal width) = 1.1; (abdominal tergite III width)/(mesonotal width) = 1.4; (forewing length)/(forewing width) = 3.2.

**Figure 3.**
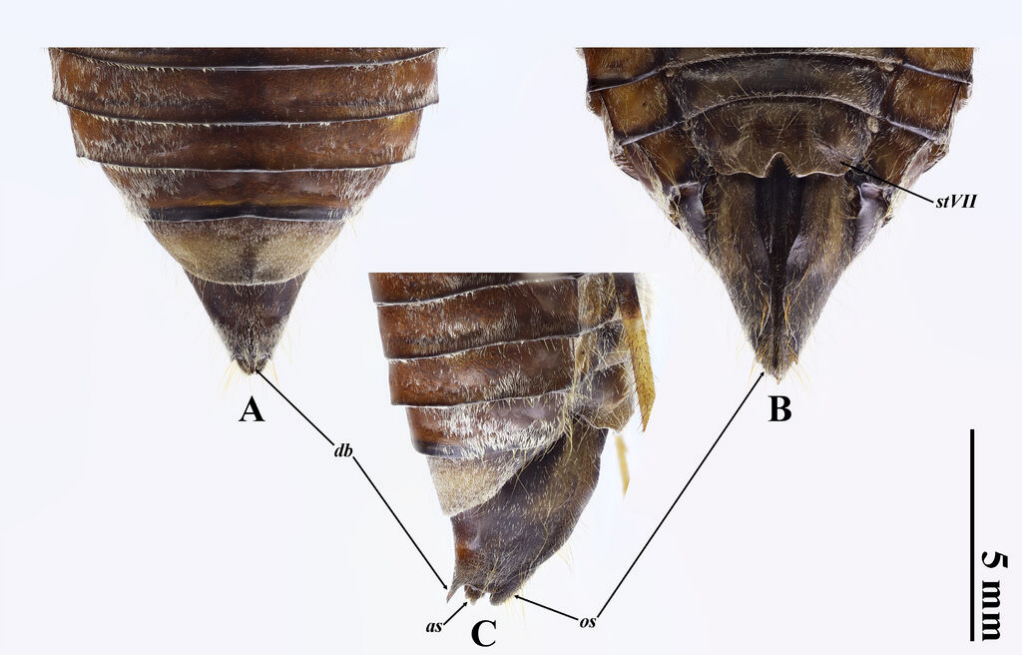
Female terminalia of *Tannafengi* Wang **sp. nov.**, paratype: **A** dorsal view; **B** ventral view; **C** lateral view. Abbreviations: as: anal styles; db: dorsal beak; stVII: sternite VII; os: ovipositor sheath.

Rostrum almost reaching middle level of abdominal sternite IV; forewing much more slender, length-width ratio 3.2 compared with 2.7 in male; abdomen considerably short, gradually narrowed in about apical half; operculum small, almost as long as wide, reaching posterior margin of abdominal sternite II; abdominal tergite III 1.4 times as wide as mesonotum; abdominal sternite VII (Fig. 3B) subtriangularly incised at middle of posterior margin and slightly protruded beside incision; ovipositor sheath blackish, not extending beyond dorsal beak; dorsal beak small, sharp, as long as anal styles.

#### Diagnosis

Lee and Emery (2020) (p. 112) stated that: "This species [*T.crassa*] is similar to *T.abdominalis* (Kato, 1938) as both species...have very wide male abdomens that are much wider than their heads and mesonota, which are not seen in any other true *Tanna* species."; likewise, *Tannafengi* Wang **sp. nov.** has a considerably wide male abdomen, which is 1.6 times as wide as mesonotum and 1.5 times as wide as head. The new species is easily distinguished from the above two closely-related species by the combination of the following characters (base on males): body length < 37.0 mm (body length also < 37.0 mm in *T.crassa*, but > 42.0 mm in *T.abdominalis*); head with blackish markings occupying most of head (head green to greenish-ochraceous or brown with normal blackish markings in both *T.abdominalis and T.crassa*); rostrum relatively longer, reaching posterior margin of abdominal sternite I (rostrum relatively shorter, extending beyond posterior margin of hind coxae in both *T.abdominalis and T.crassa*); pronotum bicoloured, fuscous at pronotal disc, while brownish at pronotal collar (pronotum bicoloured, brown at pronotal disc, while light green or greenish-ochraceous at pronotal collar in *T.abdominalis*; pronotum unicoloured, both brown at pronotal disc and collar in *T.crassa*); infuscations distinctly present on r, r-m and m crossveins, slightly on m-cu crossvein and rather inconspicuously on apices of longitudinal veins of apical cells (infuscations distinctly present on r, r-m, m and m-cu crossveins, as well as on apices of longitudinal veins of apical cells in both *T.abdominalis and T.crassa*); abdominal tergite VIII without white pollinosity (abdominal tergite VIII almost entirely covered with white pollinosity in both *T.abdominalis and T.crassa*); sternite III with paired tubercles and IV with smaller pair (only sternite III with paired tubercles in both *T.abdominalis and T.crassa*).

#### Etymology

The new species is dedicated to Mr. Lei Feng (Weifang, China), a Chinese amateur devoted to cicadas, for his help to my taxonomic study on Cicadidae. The name is a noun in the genitive case. “冯氏螗蝉” is proposed for the Chinese common name of this new species.

#### Distribution

China (Yunnan).

## Supplementary Material

XML Treatment for Tanna fengi
